# Turn Up the Heat: A Case Report of Malignant Hyperthermia During Ambulatory Surgery

**DOI:** 10.7759/cureus.61365

**Published:** 2024-05-30

**Authors:** Alexander Luong, Vincent Relli-Dempsey, Elizabeth Johnson, Dyanni Price, Andrew Gable, Matthias J Franzen

**Affiliations:** 1 Anesthesiology, OhioHealth Doctors Hospital, Columbus, USA; 2 Anesthesiology, Ross University School of Medicine, Bridgetown, BRB

**Keywords:** general anesthesia practice, anesthesia, anesthesia complication, masseter muscle rigidity after succinylcholine, risk of malignant hyperthermia, dantrolene, malignant hyperthermia (mh)

## Abstract

Malignant hyperthermia is a rare complication of general anesthesia involving the uncontrolled release of calcium when exposed to triggers such as depolarizing muscle relaxants or volatile anesthetics. It presents as a hypercatabolic skeletal muscle syndrome that results in tachycardia, hyperthermia, hypercapnia, muscle rigidity, acidosis, rhabdomyolysis, and hyperkalemia. This report presents the case of a 67-year-old female without a personal or family history of complications with anesthesia who experienced malignant hyperthermia during an elective hysterectomy. The patient was given multiple doses of dantrolene, with the ultimate resolution of her symptoms several days after surgery. She was discharged one week after surgery.

## Introduction

Malignant hyperthermia (MH) is a life-threatening autosomal dominant disorder that presents as a hypercatabolic myopathy when exposed to succinylcholine or volatile anesthetics. It has been estimated that MH episodes occur in 1:40,000 administered anesthetics, although an MH event does not occur every time MH-susceptible patients are exposed to triggering anesthetics [1]. MH occurs more frequently in children and more males than females [2]. There are specific calcium regulatory genes that are affected in this disorder: ryanodine receptor 1 (RYR1), calcium voltage-gated channel subunit alpha1 S (CACNA1S), and SH3 and cysteine-rich domain 3 (STAC3) [3,4]. Through the RYR1 gene mutation, patients susceptible to MH have abnormalities in the skeletal muscle intracellular ryanodine receptor [5]. Combined with mutations in CACNA1S and STAC3, this causes excessive intracellular calcium to be released from the sarcoplasmic reticulum leading to sustained skeletal muscle contraction [6]. This sustained contraction leads to a depletion of adenosine triphosphate and oxygen leading to the increased production of carbon dioxide and heat. These changes in metabolism also cause acidosis and a loss of cell membrane integrity leading to leakage of potassium, creatine kinase, and myoglobin [6]. Clinical manifestations include hypercapnia, hyperthermia, tachycardia, acidosis, muscle rigidity, rhabdomyolysis, hyperkalemia, myoglobinuria, and renal failure. MH is primarily a clinical diagnosis, but a confirmatory test may be done with a halothane-caffeine contracture muscle biopsy or genetic study after the event [5]. 

The mortality rate of MH without treatment is around 80% [5]. However, it has decreased over time due to the increased availability of dantrolene and the routine use of end-tidal carbon dioxide (ETCO2) monitoring. Current mortality rates have been reported to be about 5% [2]. Treating MH consists of hyperventilating the patient with 100% oxygen, discontinuing triggering agents, changing the breathing circuit/carbon dioxide absorber, and administering dantrolene sodium, a muscle relaxant that binds the RYR1 to inhibit intracellular calcium release [5]. Further treatments for this condition are supportive. 

## Case presentation

A 67-year-old female presented for elective laparoscopic robotic vaginal hysterectomy, vaginal vault suspension, bilateral salpingo-oophorectomy, and cystoscopy. She is 5'6" and weighs 115 kg, with a body mass index (BMI) of 41. Past medical history includes uterine prolapse, asthma, hypertension, urinary incontinence, and obesity. Past surgical history includes bilateral total knee arthroplasties, ultrasound breast biopsy, and a remote history of pediatric ophthalmic surgery for ophthalmic trauma. Family history was noted for coronary artery disease, stroke, and hypertension. No anesthesia complications were noted in either family or patient history. In the preoperative evaluation, the patient was afebrile (36.8°C), hypertensive (148/78 mmHg), and tachypneic (26 breaths per minute (bpm)). Preoperative complete blood count (CBC) (Table 1) and complete metabolic panel (CMP) (Table 2) performed two weeks prior were only notable for mild leukocytosis (11.19 K/mcL) and hyperglycemia (130 mg/dL). 

**Table 1:** Preoperative complete blood count

Component	Value	Reference range and units
White blood cell	11.19	4.50-11.00 K/mcL
Red blood cell	4.44	4.00-5.20 M/mcL
Hemoglobin	13.5	12.0-16.0 g/dL
Hematocrit	43.9	36.0-46.0%
Platelets	314	150-400 K/mcL

**Table 2:** Preoperative basic metabolic panel BUN: blood urea nitrogen; eGFR: estimated glomerular filtration rate

Component	Value	Reference range and units
Sodium	140	135-145 mmol/L
Potassium	4.6	3.5-5.1 mmol/L
Chloride	100	98-108 mmol/L
Bicarbonate	25	21-32 mmol/L
Anion gap	20	10-20 mmol/L
Glucose	130	65-99 mg/dL
BUN	15	8-25 mg/dL
Creatinine	0.72	0.60-1.20 mg/dL
eGFR	92	≥60 mL/min/1.73 m^2^
BUN/creatinine ratio	20.8	10.0-20.0
Calcium	9.1	8.4-10.2 mg/dL

On induction of general anesthesia, the patient received intravenous 0.5 mcg/kg fentanyl, 1 mg/kg lidocaine, 2 mg/kg propofol, and 1 mg/kg succinylcholine. She was intubated with a 7 mm cuffed oral endotracheal tube, with an initial ETCO2 reading of 44 mmHg. There was some jaw stiffness noted during intubation. Paralysis was maintained with 0.5 mg/kg rocuronium, given several minutes after intubation. Routine placement of the nasopharyngeal temperature probe revealed a temperature of 36.1°C. General anesthesia was maintained with sevoflurane. In the first hour of the procedure, the patient was mildly hypotensive with mean arterial pressures (MAP) between 60 and 65 mmHg. This was treated with boluses of intravenous fluid (IVF) and phenylephrine. ETCO2 was stable at 34-36 mmHg, and vital signs were otherwise within normal limits. At the time, the patient was ventilated on pressure-regulated, volume-controlled (PRVC) settings with an inspiratory fraction of oxygen (FiO2) of 40% at 2 L/min flow. Initial respiratory rate (RR) was 12 breaths per minute (bpm), and tidal volume (TV) was around 500 mL, approximately 4.5 mL/kg.

About four hours after the start of anesthesia, temperature was noted to have gradually increased to 38.3°C, and ETCO2 had increased to 51 mmHg. ETCO2 was noted to have increased despite increasing minute ventilation. Ventilator settings were as follows: PRVC, RR 18 bpm, and TV 600 mL. No muscle rigidity was noted. The patient had received no other neuromuscular blockers other than 160 mg succinylcholine and 50 mg rocuronium at the beginning of the case, about four hours ago. The MH hotline was contacted, and providers were told to continue monitoring the situation as there was low concern for MH at that time.

Forty-five minutes later, the temperature was noted to be 38.8°C and ETCO2 58 mmHg despite another increase in minute ventilation. Ventilator settings at this time were as follows: PRVC, RR 20 bpm, and TV 600 mL. In addition, fibroblast growth factor (FGF) had been increased to 5 L/min and FiO2 increased to 80%. The CO2 absorbent and water trap were also changed. Despite these interventions, no improvement of ETCO2 or hyperthermia was observed on monitors. Again, the MH hotline was contacted, but providers were told no dantrolene was indicated at this time. Arterial blood gas (ABG) (Table 3) was obtained to determine the patient's acid-base status.

**Table 3:** Initial arterial blood gas PaCO2: partial pressure of carbon dioxide within arterial blood; PaO2: partial pressure of oxygen within arterial blood

Component	Value	Reference range and units
pH	7.22	7.35-7.45
PaCO2	58.0	35.0-45.0 mmHg
PaO2	237	75-85 mmHg
Base excess	-4.7	-2.0-2.0
Bicarbonate	23.8	22.0-26.0 mmol/L
Ionized calcium	4.5	4.5-5.3 mg/dL
Lactic acid	5.3	0.6-2.0 mmol/L
Potassium	5.4	3.5-5.1 mmol/L

The MH hotline was contacted for a third time, and with information from the initial ABG (Table 3), providers were told to treat the case under the diagnosis of MH, and dantrolene was indicated. Sevoflurane was discontinued, and FiO2 was set to 100% at 10 L/min. Total intravenous anesthesia (TIVA) with propofol was initiated at 250 mcg/kg/min, and 2.5 mg/kg of dantrolene was given intravenously. This dose was repeated after eight minutes. A second peripheral intravenous and arterial line were placed. Ice packs were placed in the axilla. Half an ampule of HCO3 (25 mL), 0.5 g calcium chloride, 5 units of regular insulin, and 25 g dextrose 50% in water were administered. ABG was repeated (Table 4) to determine the effects of the initial treatment. An additional 5 units of regular insulin and 0.5 g calcium chloride were given. Hypotension was also noted during this time, and 60 mcg of epinephrine was given before a continuous phenylephrine infusion was started. 

**Table 4:** Repeat arterial blood gas after MH treatment MH: malignant hyperthermia; PaCO2: partial pressure of carbon dioxide within arterial blood; PaO2: partial pressure of oxygen within arterial blood

Component	Value	Reference range and units
pH	7.27	7.35-7.45
PaCO2	53.1	35.0-45.0 mmHg
PaO2	463	75-85 mmHg
Base excess	-3.0	-2.0-2.0
Bicarbonate	24.5	22.0-26.0 mmol/L
Ionized calcium	4.4	4.5-5.3 mg/dL
Lactic acid	5.1	0.6-2.0 mmol/L
Potassium	6.4	3.5-5.1 mmol/L

Within 25 minutes of the initial dantrolene bolus, the patient's temperature had improved to 37°C with an ETCO2 of 37 mmHg. At this time, the decision was made to abort the surgery and transport the patient to the intensive care unit (ICU) intubated and sedated. The maximum temperature recorded during the surgery was 39.3°C, and the maximum ETCO2 was 76 mmHg despite cooling measures and increased minute ventilation.

Upon arrival in the ICU, the serum creatine kinase (CK) level was measured to be 1056 units/L. This would ultimately peak at over 22,000 units/L (the machine could not measure greater than this) over the next two days. The electrocardiogram demonstrated normal sinus rhythm with no T-wave abnormalities. Serial CBC, CMP, ABG, CK, and lactic acid were trended every four hours till laboratory values normalized. A dantrolene infusion was started upon arrival at the ICU. On post-op day 1 (POD-1), dantrolene infusion was discontinued, and the patient was extubated.

During the ICU stay, the patient was also noted to have developed an upper extremity deep venous thrombosis and started on heparin infusion. ICU stay was otherwise uncomplicated with gradual resolution of labs and symptoms over the next several days. The patient was ultimately transferred out of ICU on POD-4. At this time, lactate had downtrended to 1.1 mmol/L and CK to 8894 units/L. The patient's hospital stay was otherwise uncomplicated, and she was discharged home on POD-7 with CK 2518 units/L and potassium 4.1 mmol/L.

On POD-13, the patient presented to the obstetrics and gynecology office for postoperative follow-up and was determined to have met postoperative milestones. During this visit, the patient had verbalized understanding of her MH event and that any children should be made aware. At the time of this publication, no muscle biopsy or genetic testing has been documented.

## Discussion

MH is an exaggerated metabolic response after exposure to either inhaled volatile agents or a depolarizing muscle relaxant such as succinylcholine [2]. This reaction typically occurs in patients with a genetic predisposition, such as alterations in genes RYR1, CACNA1S, and STAC3 [1]. Since patients susceptible to this disorder do not present with outward signs before the introduction of anesthesia, identifying patients at risk as well as diagnosis is more difficult. As previously mentioned, patients who are susceptible to MH have abnormalities in the skeletal muscle intracellular ryanodine receptor. When this receptor is mutated, there is a release of excess calcium once activation occurs by the anesthetic agent. This in turn leads to a sustained contraction of skeletal muscle, a hypermetabolic state, and increased temperature, oxygen consumption, and lactate production. The result is hypocalcemia, myoglobinuria, elevated CK, hypernatremia, and rhabdomyolysis.

MH is inherited in an autosomal dominant manner [1]. It has been estimated that 70% of cases are caused by a mutation in the RYR1 gene [2]. Most cases have been reported in young males, few have been reported in the older adult population, and it was rarely found in infants. The Upper Midwest appears to have the greatest prevalence of MH susceptibility in the United States [1].

The incidence of MH is one in 40,000 in adult patients and one in 15,000 in pediatric patients [1]. However, it should be noted that the actual prevalence can be difficult to determine due to an overall inconsistency in clinical presentation, not to mention a wide range in severity of these symptoms. Nearly 50% of patients who experience an episode of MH have had at least one previous uneventful exposure to anesthesia in which they received a triggering agent [2].

Despite the inability to use volatile anesthetics and succinylcholine, there remains a variety of pharmacologic options for the anesthesiologist. Nitrous oxide, local anesthesia, propofol, etomidate, thiopental, ketamine, opioids, and benzodiazepines have been demonstrated to be safe alternatives in MH patients (or those considered to be susceptible) [1]. Local anesthetics, both esters and amides, are also safe in MH patients [7].

MH may appear early on after the administration of anesthetic agents or later in the case. The most consistent early sign is unexplained hypercarbia despite minute ventilation that would otherwise maintain normocarbia. Other early signs of MH include muscle rigidity, increased temperature, and tachycardia. Frequent initial MH signs are hypercarbia, sinus tachycardia, or masseter spasm [2]. In the case described, the first clinical sign was masseter muscle rigidity. A refractory hypercarbia, hyperthermia, and later rhabdomyolysis all make MH the likely diagnosis despite a lack of more definitive diagnosis (e.g., muscle biopsy, genetic studies). However, in patients who demonstrate some of these features mentioned above, the anesthesiologist should also consider that these features can be present in neuroleptic malignant syndrome (NMS), serotonin syndrome (SS), and thyroid storm. 

NMS is characterized by many of the same features that mimic MH: muscle rigidity, tachycardia, and hypercarbia [4]. However, NMS is precipitated by more than just volatile anesthetics, but also by atypical antipsychotics, phenothiazines, and haloperidol [4]. These syndromes clinically can be almost indistinguishable except for the history of exposure to depolarizing muscle relaxants or inhaled anesthetics [4]. If a patient who currently has NMS is intubated, non-depolarizing muscle relaxants will result in a partial or complete resolution of muscle rigidity unlike MH [1].

SS is a drug-induced hyperthermic state that results from the administration of monoamine oxidase inhibitors (MAOI) or drugs that have selective serotonin reuptake inhibitor (SSRI) activities in a patient chronically taking an SSRI [3]. Patients will experience tachycardia, shivering, hyperreflexia, disorientation, alterations in mental status, hyperthermia, and muscle rigidity [1]. Like NMS in late stages, SS can appear similar to MH; however, the history of medications taken can distinguish between MH and SS [3].

Thyroid storm is a condition characterized by severe symptoms of thyrotoxicosis such as tachycardia, arrhythmia, cardiovascular collapse, hyperpyrexia, agitation, delirium, psychosis, and coma [8]. It is often caused by an acute event such as surgery, trauma, or infection [8]. Unlike MH, thyroid storm is typically not associated with muscle rigidity, lactic acidosis, or elevated CK [8]. Diagnosis is based on the presence of life-threatening symptoms in a patient with biochemical evidence of hyperthyroidism [8]. 

The diagnosis of MH can be presumed by using a clinical grading scale developed by Larach, with a score above 50 signifying an almost certain MH event [9]. Table 5 summarizes the clinical indicators Larach used to determine the MH raw score.

**Table 5:** Criteria used in the clinical grading scale for malignant hyperthermia

Clinical finding	Manifestation
Respiratory acidosis (15)	End-tidal CO2 >55 mmHg, PaCO2 >60 mmHg
Cardiac involvement (3)	Unexplained sinus tachycardia, ventricular tachycardia, or ventricular fibrillation
Metabolic acidosis (10)	Base deficit >8 mEq/L, pH <7.25
Muscle rigidity (15)	Generalized rigidity, severe masseter muscle rigidity
Muscle breakdown (15)	Serum creatine kinase concentration >20,000/L units, cola-colored urine, excess myoglobin in urine or serum, plasma (K+) >6 mEq/L
Temperature increase (15)	Rapidly increasing temperature, T >38.8°C
Others	Rapid reversal of malignant hyperthermia signs with dantrolene (score=5), elevated resting serum creatine kinase concentration (score=10)
Family history (15)	Consistent with autosomal dominant inheritance

The initial treatment is to discontinue any volatile agents and hyperventilate the patient with 100% oxygen to counteract the effects of increased carbon dioxide and increased oxygen consumption. The breathing circuit and carbon dioxide absorber should be changed. If the surgery cannot be aborted, anesthesia should be maintained with total intravenous anesthesia or other methods that avoid inhaled volatile anesthetics. The drug of choice for treatment is dantrolene, which will decrease the intracellular calcium availability and slow down muscle contraction. The initial dose is 2.5 mg/kg intravenously every five minutes until the clinical signs are controlled [2]. After initial control of the symptoms, the patient should be transferred to the ICU for at least 24 hours, and 1 mg/kg of dantrolene should be given intravenously every six hours for 24-48 hours to prevent relapse [1].

As discussed above, hyperkalemia and rhabdomyolysis commonly present later in the disease course, commonly alongside lactic acidosis. These should be corrected with supportive measures, including intravenous fluids and calcium salts. CK levels should be trended during this time also until rhabdomyolysis has clinically resolved. Dantrolene has been associated with an acute liver injury [10]. Therefore, it is also recommended that liver enzymes be trended.

## Conclusions

This case demonstrates an ambiguous presentation of MH in an elective outpatient obstetric surgery. Although the patient did not present with generalized muscle rigidity, as is common in MH, some masseter muscle rigidity was noted during intubation. This illustrates the necessity of having a low threshold to diagnose MH intraoperatively. Early identification and treatment are imperative for a favorable outcome in patients who experience MH. In suspected cases, the provider should discontinue all volatile anesthetics and switch to total intravenous anesthesia. A bolus of dantrolene 2.5 mg/kg should be administered and repeated until clinical improvement. In this case, treatment was delayed until the third phone call with the MH hotline. Fortunately, the patient survived. It is important for providers to recognize the different nuanced presentations of MH contrasted with similar disorders. We present this case with the intention of helping other clinicians develop a higher suspicion of MH when in a similar situation.
